# PPARD May Play a Protective Role against the Development of Schizophrenia

**DOI:** 10.1155/2020/3480412

**Published:** 2020-08-07

**Authors:** Xinrong Li, Sha Liu, Karan Kapoor, Yong Xu

**Affiliations:** ^1^Shanxi Key Laboratory of Artificial Intelligence Assisted Diagnosis and Treatment for Mental Disorder, First Hospital of Shanxi Medical University, Taiyuan, China; ^2^Department of Psychiatry, First Hospital/First Clinical Medical College of Shanxi Medical University, Taiyuan, China; ^3^NIH Center for Macromolecular Modeling and Bioinformatics, Beckman Institute for Advanced Science and Technology, University of Illinois at Urbana-Champaign, Urbana, IL 61801, USA

## Abstract

PPARD has been suggested to contribute to the etiology of schizophrenia (SCZ) with the underlying mechanisms largely unknown. Here, we first collected and analyzed the PPARD expression profile from three groups: (1) 18 healthy control (HC) subjects, (2) 14 clinical high-risk (CHR) patients, and (3) 19 early onset of SCZ (EOS) patients. After that, we conducted a systematical pathway analysis to explore the potential mechanisms involved in PPARD exerting influence on the pathological development of SCZ. Compared to the HC group, the expression of PPARD was slightly decreased in the EOS group (LFC = −0.34; *p* = 0.23) and increased in the CHR group (LFC = 0.65; *p* = 0.20). However, there was a significant difference between the EOS group and the CHR group (LFC = −0.99; *p* = 0.015), reflecting the amount of variation in PPARD expression before and after the onset of SCZ. Pathway analysis suggested that overexpression of PPARD may regulate ten proteins or molecules to inhibit the pathological development of SCZ, including the deactivation of eight SCZ promoters and stimulation of two SCZ inhibitors. Our results support the association between PPARD and SCZ. The pathways identified may help in the understanding of the potential mechanisms by which PPARD contributes to the etiology of SCZ.

## 1. Introduction

Schizophrenia (SCZ) is a common and often disabling mental illness characterized not only by a varied group of clinical symptoms [1], but wide-ranging deficits in neurocognitive and neurophysiological functions [2, 3]. The prodromal period is thought to have a high risk of clinical symptoms and precedes illness onset by 1 to 6 years [4, 5]. Subjects with these characteristics are called clinical high-risk (CHR) patients, with about one-third developing SCZ and two-third recovering to normal [4, 5] (PMID: 8782291; PMID: 1571314). Early-onset SCZ (EOS), defined as SCZ with onset before the 21st birthday, shows worse psychosocial disability and poor prognosis [6]. As a neurodevelopment disease [7], SCZ in childhood tends to have a higher possibility of abnormal neural development [8].

PPARD is a nuclear hormone receptor that governs a variety of biological processes [9]. This gene has been suggested to play roles in the development of several chronic diseases, including diabetes, obesity, atherosclerosis, and cancer [10]. Several studies have suggested that PPARG may contribute to the etiology of SCZ [11–13]. For instance, Sun et al.'s study showed that the PPARD polymorphism rs2076169 had an allelic association with SCZ (X2 = 13.62, *p* = 0.0002) in a trio study using a transmission disequilibrium test [11]. Maekawa et al. identified a significantly downregulated expression of PPARD (*p* < 0.05) in individuals with SCZ compared with the control subjects [12]. Dzana et al. also discovered linkages between the genetic variants of multiple genes including PPARD and the increased waist circumference in SCZ patients (*p* < 0.037) [13]. However, the underlying mechanism regarding the PPARD-SCZ association is largely unknown.

To explore the relationship between PPARD and SCZ, we studied the expression changes of PPARD in both CHR and EOS groups and compared that to the healthy control (HC) group. CHR is a special state of SCZ before its onset. We hypothesized that the changes in PPARD expression in the CHR group could lead to the regulation of genes not observed in the SCZ group. After that, we conducted a systematic bioinformatics analysis and identified multiple pathways through which PPARD could exert influence on SCZ. Our study provides novel evidence for the association between PPARD and SCZ and adds new insights into the understanding of the roles of PPARD in the etiology of SCZ.

## 2. Materials and Methods

### 2.1. Subject Recruitment for Expression Profile Collection

All participants were unrelated Han Chinese recruited from the north of China and under the age of 18 years. EOS patients were diagnosed by 2 associate doctors according to the Diagnostic and Statistical Manual of Mental Disorders: Fourth Edition (DSM-IV) and the Chinese version of the Modified Structured Clinical Interview for DSM-IV, patient version (SCID-I/P). The total score of PANSS was ≥60, and IQ score was ≥70. Exclusions included patients with organic disease of heart, liver, and kidney; all kinds of immune diseases, brain injury, or brain congenital malformation; a tumor of brain and epilepsy; mental retardation, along with anyone taking antipsychotic drugs, antimanic drugs, antidepressants, or mood stabilizers. In addition, we excluded serious excitement or impulsion patients. CHR patients were assessed with the Structured Interview of Prodromal Syndromes (SIPS) [14]. The exclusion criteria of this group were the same as the EOS group. The healthy controls (HC) were selected by matching the age and sex with never having taken any drugs in the latest one month. Exclusion criteria included (1) meeting the standards of inclusion or exclusion term of patients, (2) having the family history of any spirit or nervous system disease, (3) having head injury or newborn related disease, (4) having hyperpyretic convulsion before, and (5) being an adopted child or living in a single-parent family.

All teenage participants' informed consent was signed by their parents. The study was conducted under the protocols approved by the First Hospital of Shanxi Medical University (Ethical Code: 2019-Y01).

### 2.2. RNA Extraction and Quantity Control

Total RNA was extracted from all of the samples which had been snap-frozen using TRIzol reagent (Invitrogen, Carlsbad, CA, U.S.) according to the manufacturer's previous protocol [15]. Total RNA from each sample was quantified by the NanoDrop ND-1000, and RNA integrity was assessed by standard denaturing agarose gel electrophoresis.

### 2.3. RNA Labeling and Microarray Hybridization

The RNA labeling and microarray hybridization followed the routine process described as follows [16]. The Arraystar Human LncRNA Array v2.0 is designed for researchers who are interested in profiling both lncRNAs and protein-coding RNAs in the human genome. Sample labeling and array hybridization were performed according to the Agilent One-Color Microarray-Based Gene Expression Analysis protocol (Agilent Technology) with minor modifications. Briefly, mRNA was purified from total RNA after removal of rRNA (mRNA-ONLY™ Eukaryotic mRNA Isolation Kit, Epicentre). Then, each sample was amplified and transcribed into fluorescent cRNA along the entire length of the transcripts without 3′ bias utilizing a random priming method. The labeled cRNAs were purified by the RNeasy Mini Kit (Qiagen). The concentration and specific activity of the labeled cRNAs (pmol Cy3/*μ*g cRNA) were measured by NanoDrop ND-1000. 1 *μ*g of each labeled cRNA was fragmented by adding 11 *μ*l 10x Blocking Agent and 2.2 *μ*l of 25x Fragmentation Buffer and then heating the mixture at 60°C for 30 min. Finally, 55 *μ*l 2x GE Hybridization buffer was added to dilute the labeled cRNA. 100 *μ*l of hybridization solution was dispensed into the gasket slide and assembled on the RNA expression microarray slide. The slides were incubated for 17 hours at 65°C in an Agilent Hybridization Oven. The hybridized arrays were washed, fixed, and scanned using the Agilent DNA Microarray Scanner (part number G2505C). The microarray work was performed by KangChen Bio-tech (Shanghai).

### 2.4. Bioinformatics Analysis

To gain a better understanding of the gene expression resulting from different groups of subjects and explore possible roles of PPARD in the etiology of SCZ, we conducted a literature-based pathway analysis to identify the possible molecular pathways connecting PPARD and SCZ. Specifically, by using the tool Pathway studio (version 12.3; http://www.pathwaystudio.com), we identified genes and small molecules that are downstream targets of PPARD and upstream regulators of SCZ with polarity. Then, we constructed the PPARD-driven functional pathways with polarity and direction.

## 3. Results

### 3.1. Demographics

The three groups of subjects were comparable in age and gender. In all, we recruited 19 EOS patients (8 males and 11 females, aged 14.79 ± 1.90 years), 14 CHR patients (9 males and 5 females, aged 16.14 ± 1.41 years), and 18 HC (9 males and 9 females, aged 15.67 ± 2.40 years). The demographic information for all participants is provided in Table 1.

### 3.2. Expression Variation of PPARD in Different Groups

**Figure 1:**
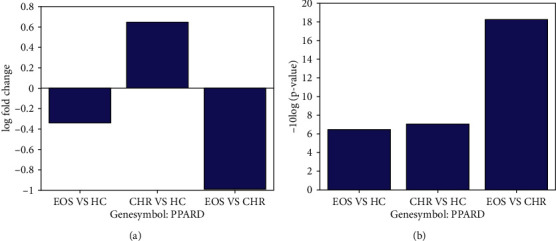
PPARD expression comparison among three different groups: healthy control (HC) group, clinical high-risk (CHR) group, and early-onset schizophrenia (EOS) group. (a) The -10 ∗ log10 transferred *p* values in different comparisons; (b) the log fold change of PPARD expression for different comparisons.

Compared with the HC group, PPARD presented increased expression levels in the CHR group (LFC = 0.65) and decreased expression levels in the EOS group (LFC = −0.34), as shown in Figure 1(a). The changes were milder in terms of the statistical *p* value (*p* value = 0.23 and 0.20 for EOS vs HC and CHR vs HC, respectively). However, the difference between EOS and CHR group showed statistical significance (*p* value = 0.015; LFC = −0.99). The downregulation of PPARD in the EOS group was consistent with previous study results (PubMed 28872641); however, the increased PPARD expression levels in the CHR groups were not reported before. Usually, there are about two-third of the CHR subjects who do not develop SCZ (PMID: 8782291; PMID: 1571314). Thus, our results indicated that increased PPARD expression might play a protective role against the development of SCZ, with the underlying mechanism explored using subsequent pathway analysis.

### 3.3. Genetic Pathways Driven by PPARD

**Figure 2:**
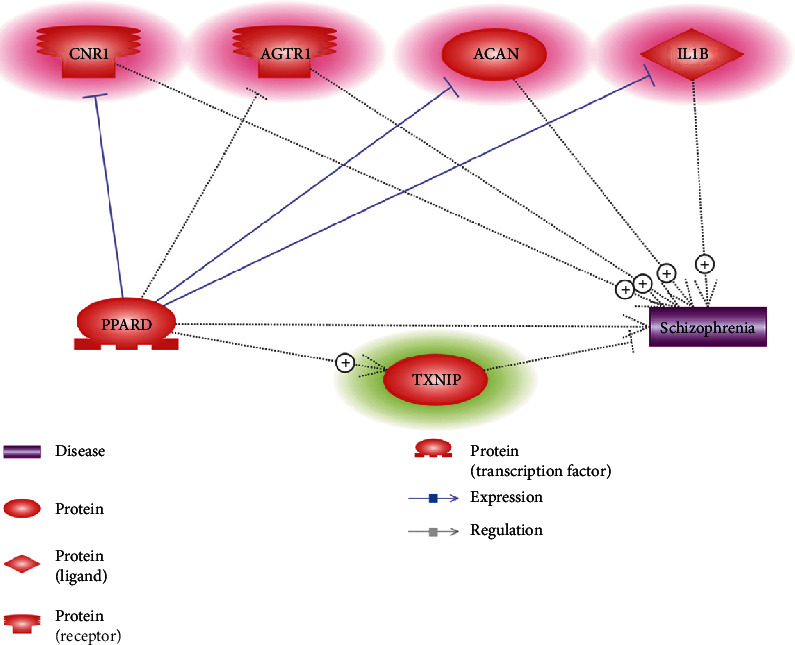
PPARD driven genetic pathways inhibiting schizophrenia. The genes highlighted in red are schizophrenia promoters; green denoting schizophrenia inhibitors.

To understand the possible roles that PPARD could exert on the pathologic development of SCZ, we constructed a literature-based genetic pathway connecting PPARD and SCZ, as shown in Figure 2. Our results showed that PPARD deactivates four promoters of SCZ, including CNR1, AGTR1, ACAN, and IL1B. Moreover, one SCZ inhibitor could also get activated by PPARD. These results may partially explain the mechanism regarding the roles of PPARD in the etiology of SCZ. Each relation within Figure 2 was supported by one or more scientific references (Supplementary Material: Ref 4Figure 2). The corresponding sentences where a relationship has been identified were reviewed to confirm the confidence of the identified relation.

### 3.4. Molecule Pathways Driven by PPARD

**Figure 3:**
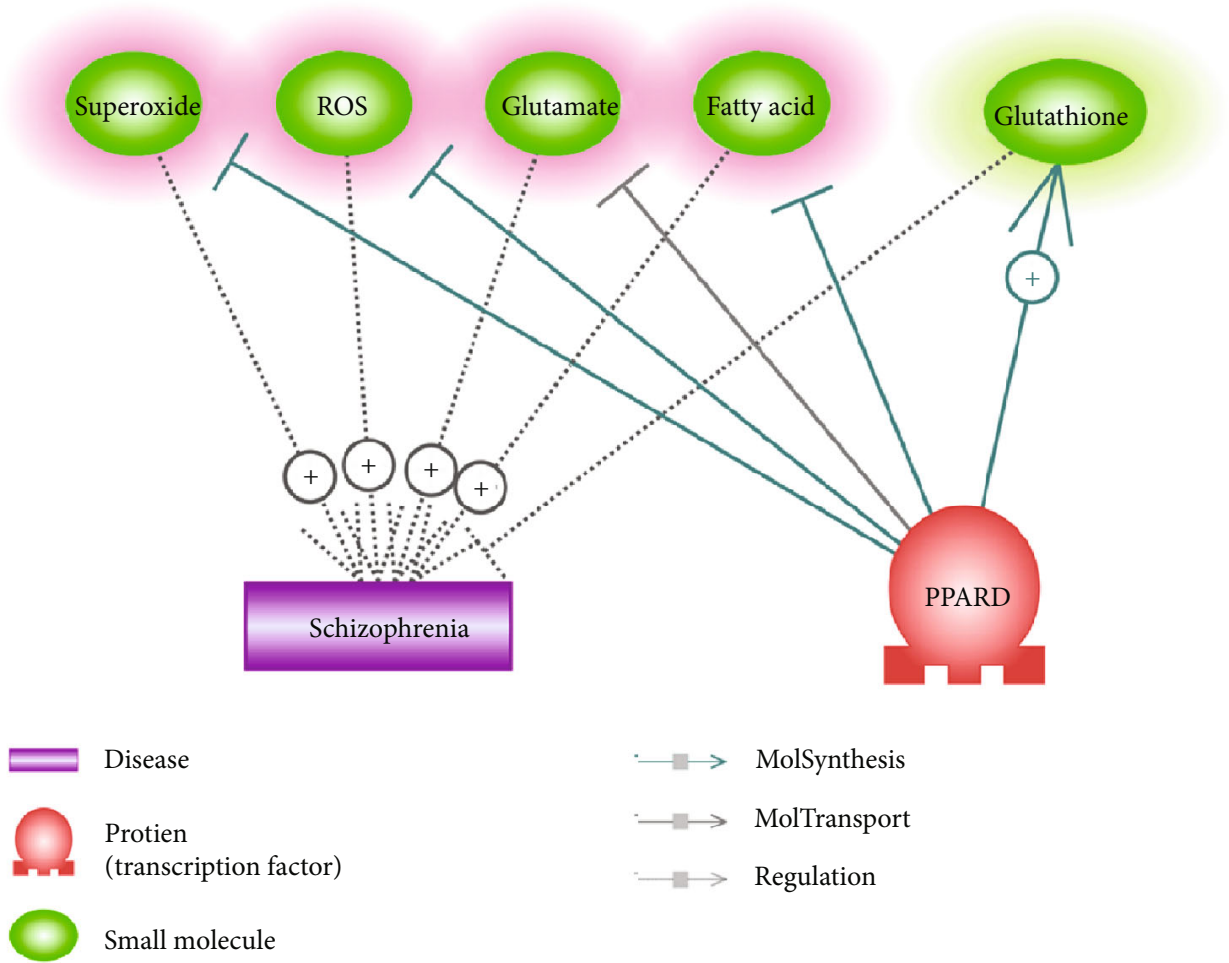
PPARD driven genetic pathways inhibiting schizophrenia. The molecules highlighted in red are schizophrenia promoters; green denoting schizophrenia inhibitors.

Besides the genetic pathway, we also identified multiple molecules influencing the pathologic development of SCZ and regulated by PPARG, as shown in Figure 3. Specifically, PPARG promotes the secretion of glutathione, which is an inhibitor of SCZ. Moreover, PPARD suppresses four molecules that could promote the development of SCZ, including superoxide, ROS, glutamate, and fatty acid. These results may further explain the underlying mechanism in which increased expression of PPARD could protect against the development of SCZ. Each relation within Figure 3 was supported by one or more scientific references (Supplementary Material: Ref 4Figure 3). The corresponding sentences where a relationship has been identified were reviewed to confirm the confidence of the identified relation.

## 4. Discussion

Previous studies have suggested that PPARD may contribute to the etiology of SCZ. However, the underlying mechanism is largely unknown [11, 12]. To better understand the role of PPARD, we tested the expression variation of PPARD in both CHR and EOS groups and compared it to the HC group. We hypothesize that PPARD expression changes in the CHR group may reflect the activities of genes during the development of SCZ but before its onset. Our results showed that the EOS group presented decreased expression levels compared to healthy controls, which was consistent with a previous study [12]. However, we observed increased PPARD expression in the CHR group compared to both HC and EOS groups. The opposite change between CHR and EOS groups may reflect the PPARD variation before and after the onset of SCZ.

Pathway analysis showed that increased expression of PPARD might drive proteins and small molecules to protect against the pathologic development of SCZ, as shown in Figures 2 and 3. These pathways suggested that increased expression of PPARD could inhibit SCZ promoters and activate SCZ inhibitors, consequently influencing the pathophysiology of SCZ. For instance, PPARD has been shown to inhibit CB1 receptor expression (CNR1), which contributes to the pathophysiology of SCZ [17]. PPARD also reduces the upregulation of angiotensin II type 1 receptor (AGTR1) [18], the antagonists of which have been reported to improve clinical symptoms in SCZ patients [19]. In addition, PPARD activation promotes the degradation of aggrecan (ACAN) and attenuates gene expression of IL1B [20, 21]. Both ACAN and IL1B were suggested to contribute to the increased risk of SCZ [22, 23]. Moreover, SCZ patients have been shown to present decreased expression of tumor suppressor gene TXNIP, a thioredoxin-binding protein that is a member of the alpha arrestin protein family [24]. Ratneswaran et al. showed that PPARD could upregulate the expression of TXNIP [25]. These results support the association between PPARD and SCZ and also suggest the possible mechanisms of the protective roles of PPARD in the pathologic development of SCZ.

Our study also shows that PPARD may inhibit the generation and release of four molecules that play important roles in the pathophysiology of SCZ, including superoxide, oxygen free radicals (ROS), glutamate, and fatty acid (Figure 3). Moreover, it has been shown that agonists of PPARD could promote glutathione synthesis [26], with the deficit of glutathione impairing neurotransmission and cerebral connectivity that lead to clinical symptoms of SCZ [27]. These molecule pathways add more support for the mechanisms involved in the PPARD-SCZ association.

To our knowledge, no previous study has explored the PPARD expression variations between CHR patients and the EOS group, which provide new vision of the activity of PPARD in the case of SCZ. This study also has some limitations. Firstly, larger datasets could be collected to validate the expression levels of PPARD in CHR and EOS patients. Secondly, a follow-up study should be conducted to identify the expression of PPARD throughout the clinical high-risk stage to the recovery or disease stage of SCZ. Due to the limitation of supporting sources, this work has been left for future studies.

## 5. Conclusion

Our results confirmed decreased expression of PPARD in the case of SCZ and revealed increased expression in the clinical high-risk group. Pathway analysis suggested that the overexpression of PPARD in the high-risk group may contribute to the protection of a subject from developing SCZ.

## Figures and Tables

**Table 1:** Demographics and clinical characteristics for all participants.

	EOS patients	CHR patients	HC	*F/x* ^2^	*p* value
*n*	19	14	18		
Age (years)	14.79 ± 1.90	16.14 ± 1.41	15.67 ± 2.40	2.012	0.145
Gender (M/F)	8/11	9/5	9/9	1.598	0.450
PANSS total scores	62.17 ± 13.32				

Note: PANSS; Positive and Negative Syndrome Scale.

## Data Availability

The data of this study are available from the corresponding author upon reasonable request.

## Supplementary Materials

Supplementary Materials | Reference for Figure 2 and reference for Figure 3.
